# Pooled DNA sequencing to identify SNPs associated with a major QTL for bacterial wilt resistance in Italian ryegrass (*Lolium multiflorum* Lam.)

**DOI:** 10.1007/s00122-018-3250-z

**Published:** 2018-11-30

**Authors:** Verena Knorst, Stephen Byrne, Steven Yates, Torben Asp, Franco Widmer, Bruno Studer, Roland Kölliker

**Affiliations:** 1 Molecular Plant Breeding, Institute of Agricultural Sciences, ETH Zürich, Universitätsstrasse 2, 8092 Zurich, Switzerland; 2 Molecular Ecology, Agroscope, Reckenholzstrasse 191, 8046 Zurich, Switzerland; 3 Crops Science Department, Teagasc, Oak Park, Carlow, R93 XE12 Ireland; 4 Department of Molecular Biology and Genetics, Section for Crop Genetics and Biotechnology, Forsøgsvej 1, 4200 Slagelse, Denmark

## Abstract

**Key message:**

SNPs and candidate genes associated with bacterial wilt resistance in Italian ryegrass were identified by sequencing the parental plants and pooled *F*_1_ progeny of a segregating population.

**Abstract:**

Italian ryegrass (*Lolium multiflorum* Lam.) is one of the most important forage grass species in temperate regions. Its yield, quality and persistency can significantly be reduced by bacterial wilt, a serious disease caused by *Xanthomonas translucens* pv. *graminis*. Although a major QTL for bacterial wilt resistance has previously been reported, detailed knowledge on underlying genes and DNA markers to allow for efficient resistance breeding strategies is currently not available. We used pooled DNA sequencing to characterize a major QTL for bacterial wilt resistance of Italian ryegrass and to develop inexpensive sequence-based markers to efficiently target resistance alleles for marker-assisted recurrent selection. From the mapping population segregating for the QTL, DNA of 44 of the most resistant and 44 of the most susceptible *F*_1_ individuals was pooled and sequenced using the Illumina HiSeq 2000 platform. Allele frequencies of 18 × 10^6^ single nucleotide polymorphisms (SNP) were determined in the resistant and susceptible pool. A total of 271 SNPs on 140 scaffold sequences of the reference parental genome showed significantly different allele frequencies in both pools. We converted 44 selected SNPs to KASP™ markers, genetically mapped these proximal to the major QTL and thus validated their association with bacterial wilt resistance. This study highlights the power of pooled DNA sequencing to efficiently target binary traits in biparental mapping populations. It delivers genome sequence data, SNP markers and potential candidate genes which will allow to implement marker-assisted strategies to fix bacterial wilt resistance in outcrossing breeding populations of Italian ryegrass.

**Electronic supplementary material:**

The online version of this article (10.1007/s00122-018-3250-z) contains supplementary material, which is available to authorized users.

## Introduction

High-yielding forage grasses are major components of temperate grasslands worldwide, providing an important feed source for sustainable ruminant livestock production (Humphreys et al. 2010). Italian ryegrass (*Lolium multiflorum* Lam.) is one of the most important forage grasses, mainly used for hay and silage production and particularly valued for its high palatability and biomass yield (Bernard et al. 2002). Biomass yield of Italian ryegrass can be severely reduced by various bacterial and fungal pathogens. The most important bacterial pathogen of Italian ryegrass is *Xanthomonas translucens* pv. *graminis* (Xtg), which causes bacterial wilt on a range of forage grass species including ryegrasses and fescues (Egli et al. 1975). Yield losses are reported to range between 20% in field swards (Schmidt and Nuesch 1980) and up to 80% in experimental setups after leaf inoculation (Wang and Sletten 1995). Xtg is a gram-negative bacterium of the genus *Xanthomonas*, which harbors other economically important pathogens such as *X. oryzae* pv. *oryzae* (Xoo) causing blight in rice (Duku et al. 2016) and *X. campestris* pv. *campestris* (Pammel) Dowson causing black rot in cruciferous vegetables (Singh et al. 2011).

In contrast to fungal pathogens, which reproduce mainly on the plant’s surface, Xtg enters its host via wounds or leaf stomata, multiplies in the xylem (Chan and Goodwin 1999) and is transmitted by contaminated mowing equipment (Schmidt 1988). As a bacterial disease, chemical protection against Xtg is difficult and would involve the application of antibiotics which is not tolerated in grasslands and bears the risk of resistance formation (Tancos et al. 2015). Therefore, breeding for resistant cultivars is the only ecologically and economically feasible method to control the disease. This makes resistance against Xtg to one of the major breeding goals in Italian ryegrass.

Breeding strategies to improve Xtg resistance are based on the selection of parental plants displaying various sources of resistance against Xtg using artificial inoculation (Boller and Lehmann 1996). This resistance screening is time- and resources-consuming. A marker-assisted selection (MAS) strategy may not only allow for shorter selection time, but also it has the additional advantage to distinguish plants homo- or heterozygous for the resistance allele. This is of particular interest for allogamous species such as Italian ryegrass, which are mainly bred as populations (Posselt 2010). Due to the high level of heterozygosity, dominant resistance alleles may mask the presence of susceptibility alleles, which are therefore maintained in phenotypically selected breeding populations. Upon recombination, homozygosity of susceptibility alleles can occur and cause the emergence of susceptible plants, even in advanced breeding cycles (Acquaah 2012; Michel 2001).

A better understanding of the genetic basis governing the resistance mechanism and the host–pathogen interaction is required to establish efficient MAS-based resistance breeding strategies. Generally, plant immune systems are based on two response layers and can be described by the zig-zag-model (Jones and Dangl 2006). The first layer, pathogen-associated molecular pattern (PAMP) triggered immunity (PTI), is based on the detection of pathogens at the plant’s surface by pathogen recognition receptors (PRRs). If the pathogen is able to overcome this defense layer by introducing effector molecules into the plant cell, often via the type III secretion system, the pathogen is able to infect the plant leading to effector-triggered susceptibility (ETS; Pavan et al. 2009). As this arm race continues, plants can detect these effectors with resistance (R) genes and launch a strong defense reaction upon recognition, i.e., effector-triggered immunity (ETI). The fact that on average about 2% of the annotated genes in plant genomes encode for R genes and that these genes are often arranged in clusters, facilitating the inheritance of the whole set, emphasizes the importance of these genes (Sekhwal et al. 2015). So far, resistance genes against *Xanthomonas* spp. have been identified in various crop species. For example in rice (*Oryza sativa* L.), seven major resistance genes against Xoo (*Xa1*, *Xa3/Xa26*, *xa5*, *xa13*, *Xa21*, *xa25*, *Xa27*) have been characterized (reviewed in Zhang and Wang 2013). The gene *Xa21* is a typical R gene, encoding for a receptor kinase-like protein, with a leucine-rich repeat motif and a serine–threonine kinase-like domain (Song et al. 1995). Interestingly, *Xa21* has also been shown to act as a pattern recognition receptor for the PAMP effector *Ax21* (reviewed in Liu et al. 2013). The recessive R gene *xa25* encodes for OsSWEET13 (Sugars Will Eventually be Exported Transporters), interacts with the transcription activator-like (TAL) effector PthXo1 and is therefore a typical example for ETI (Chen 2014). In pepper (*Capsicum* spp.) and tomato (*Solanum lycopersicum* L.), four different *Xanthomonas* spp. were identified causing bacterial spot symptoms, *X. vesicatoria*, *X*. *euvesicatoria*, *X. perforans* and *X. gardneri* (Stall et al. 2009). A number of different resistance genes and their corresponding effectors were described for that pathosystem (Stall et al. 2009).

A first step toward understanding bacterial wilt resistance in Italian ryegrass was the development of a biparental mapping population (hereafter referred to as Xtg-ART), segregating for a major resistance source (Studer et al. 2006). With that population, a genetic linkage map comprising 367 AFLP and 51 SSR markers on seven linkage groups (LGs) was established and used for QTL mapping. A major QTL explaining up to 84% of the observed phenotypic variation was identified on LG 4. However, further characterization of the QTL and the development of closely linked markers to be used in breeding programs were hindered by the predominantly anonymous AFLP markers associated with the resistance and the limited genome sequence information available for Italian ryegrass at the time.

Recently, a draft genome sequence of Italian ryegrass has been established by shotgun sequencing of the resistant parental plant of the Xtg-ART mapping population, consisting of 5.74E + 08 bp in 129,579 scaffolds (N50 4949 bp), corresponding to a 28 X coverage (Knorst et al. 2018). Although this draft assembly of the low-copy fraction of the genome is still very fragmented, it provides an excellent basis for marker development to further uncover the major QTL on LG 4. Bulked segregant analysis (BSA) using high-coverage DNA sequencing and subsequent SNP allele frequency analysis provide an efficient tool to screen a large number of plants to identify SNPs associated with the trait under study (Michelmore et al. 1991; Schneeberger 2014; Zou et al. 2016).

The main objective of this study was to characterize a previously reported major QTL for bacterial wilt resistance on LG 4 in Italian ryegrass by next-generation sequencing (NGS) and BSA using the biparental mapping population Xtg-ART. Specifically, we aimed at (i) providing sequence information of the parental plants of Xtg-ART and their most resistant and susceptible offspring, (ii) identifying SNP markers co-segregating with Xtg resistance and (iii) using these SNPs for the identification of candidate genes underlying the Xtg resistance in Italian ryegrass and to provide an inexpensive marker system for deployment in resistance breeding.

## Materials and methods

### Sequencing of the parental plants and pools of *F*_1_ individuals in Xtg-ART

DNA from the parental and offspring plants of the biparental mapping population Xtg-ART, developed for characterization of bacterial wilt resistance in Italian ryegrass (Studer et al. 2006), was extracted using the DNeasy plant 96 kit (Qiagen, Hilden, Germany). DNA quality was assessed by agarose gel electrophoresis and quantified through spectrophotometry (NanoDrop; Thermo Fisher Scientific, Waltham, Massachusetts, USA).

To complement the already described draft genome sequence of the resistant Xtg-ART parent M2289 (Knorst et al. 2018), the genome of the susceptible Xtg-ART parent Adret2 was sequenced de novo on the Illumina HiSeq 2000 platform (Illumina Inc., San Diego, USA) using 100 bp paired-end reads of fragment libraries with insert sizes of 300 and 800 bp. ABySS (Version: 1.3.5; Simpson et al. 2009) was used for sequence assembly with a k-mer size of 55 bp, raw data from sequencing of both libraries (300 and 800 bp), a minimum base quality of 30 and a minimum contig size of 300 bp to build scaffolds with data from the larger insert library. To get a quantitative measure of the completeness of the gene space, the Benchmarking Universal Single-Copy Orthologues (BUSCO, V3) gene set with the Embryophyta odb9 lineage was searched, using the following specifications: creation date 2016-02-13, number of species 30 and number of BUSCOS 1440 (Simão et al. 2015).

**Fig. 1:**
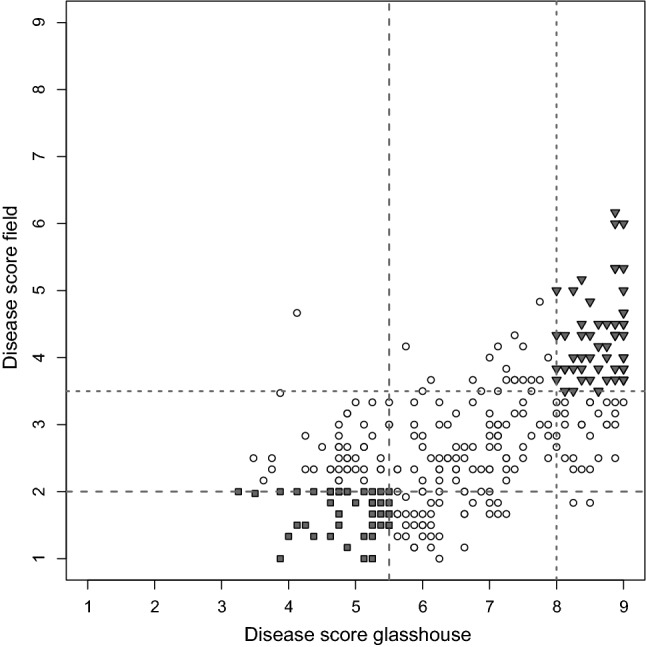
Selection of Italian ryegrass *F*_1_ individuals of the Xtg-ART mapping population for susceptible and resistant pools based on disease scores previously reported for a glasshouse and a field experiment (Studer et al. 2006). Disease scores range from 1 = resistant to 9 = susceptible. Resistant (gray squares) and susceptible (gray triangles) individuals were selected based on the upper (dotted line) and lower (dashed line) quartile of the scores from the field and the glasshouse experiment

*F*_1_ individuals of Xtg-ART were classified as susceptible or resistant based on the disease scores reported by Studer et al. (2006) and obtained in the field or in the glasshouse using the isolate Xtg29. For classification, the upper and lower quartiles of the scores obtained in both experiments (corresponding to disease scores of 8.0 and 5.5 in the glasshouse and 3.5 and 2.0 in the field) were used (Fig. 1). Based on availability and disease scores, 44 susceptible and 44 resistant individuals were selected for DNA extraction and 0.45 µg of DNA from each individual was pooled to form the susceptible and the resistant pool, respectively. Sequencing libraries were prepared using the NEBNext DNA sample kit (New England Biolabs, Ipswich, Massachusetts, USA) with Illumina adaptors according to the NEBNext instructions. Both pools were sequenced with 100 bp paired-end reads on the Illumina HiSeq 2000 platform (Illumina Inc., San Diego, USA).

### Discovery of SNPs segregating in Xtg-ART

To identify SNPs segregating in the Xtg-ART mapping population (hereafter referred to as “SegSNPs”), raw sequencing reads of the susceptible (Adret2) and the resistant parental plant (M2289) were realigned to the M2289 de novo assembly, which is currently the only publicly available reference assembly of Italian ryegrass (Knorst et al. 2018). Genotypes in each sample were called using a minimum Genotype Quality (GQ) threshold of 30 (Phred scale); below, data points were recorded as missing. SNPs were identified and filtered to only include (i) those heterozygous in at least one parental genotype, (ii) biallelic SNPs and (iii) those with a minimum mapping quality of 30 (Phred scale), using the UnifiedGenotyper of the Genome Analysis Tool Kit (GATK, Broad Institute, Cambridge, MA, USA).

### Sequence analysis of the *F*_1_ pools

Sequencing reads from each of the two pools were realigned to the M2289 de novo assembly (Knorst et al. 2018) using the Burrows–Wheeler Aligner (BWA; Li and Durbin 2009) and only those sites that were previously identified as ‘SegSNPs’ and contained a minimum of 20 reads per pool were selected. At each position, SNP allele frequencies were analyzed using fixation index (*F*_ST_) values, calculated with PoPoolation (Kofler et al. 2011). Genome scaffolds of the resistant parental plant M2289 containing at least one SNP with a *F*_ST_ value of ≥ 0.9 were selected and compared to the synteny-based draft genome assembly of perennial ryegrass (Byrne et al. 2015) by BLAST analysis. The genome position of the corresponding perennial ryegrass scaffolds was determined using the perennial ryegrass GenomeZipper (Pfeifer et al. 2013).

### SNP genotyping, marker validation, linkage map and QTL analysis

SNPs identified as putatively associated with disease resistance above were selected for conversion to competitive allele specific PCR (KASP™) assays followed by validation via genetic mapping within the Xtg-ART mapping population. A total of 221 *F*_1_ individuals from the Xtg-ART mapping population were genotyped with the newly developed KASP™ markers (LGC Genomics Ltd., Hertfordshire, UK). Single-marker linear regression using individual marker scores and the phenotypic data from the glasshouse and two field seasons reported by Studer et al. (2006) were used to validate the association of individual markers with bacterial wilt resistance. For this, markers were coded either 1 (homozygous reference allele of the KASP™ assay), 2 (heterozygous reference/variant allele) or 3 (homozygous variant allele) and *R*^2^ and *P* values (not corrected for multiple testing) were reported. Statistical analyses were performed using the R software environment (R Core Team 2018).

KASP™ data were recoded for import into JoinMap 4.1 (Van Ooijen and Voorrips 2001) and used to calculate the location of the SNP markers and the corresponding M2289 scaffolds in the region of the major QTL on LG 4. In order to consolidate marker positions relative to the QTL, six AFLP markers flanking (E39M50_127, E38M59_300, P35M59_183, E32M48_127, P32M49_171) or co-locating (P38M50_252) with the major QTL in the original study (Studer et al. 2006) were included in map calculation with their original genotyping data. Kosambi’s mapping function for regression mapping was used to calculate the maps. Maps for the two parental genotypes were calculated separately and combined using marker located on both maps.

For QTL analysis, the MQM algorithm of MapQTL6 was used (Van Ooijen 2009). Cofactors were selected with the function “Automatic Cofactor Selection”. The thresholds for significant correlation between marker and the disease resistance trait were calculated with a permutation test using 5000 iterations.

To simulate the potential success of MAS, KASP markers with a *R*^*2*^ values ≥ 0.2 from single-marker regression and mapping to LG 4 were selected. For each marker or combinations of two or three markers, mean disease scores obtained in the glasshouse for individuals homozygous for the allele(s) associated with resistance (positive selection) and for individuals homozygous for the alternative allele(s) (negative selection) were calculated and compared to the mean values of the entire mapping population.

## Results

### Bulked segregant analysis (BSA) in Xtg-ART

For a comprehensive overview of the genome constitution in the parental plants of Xtg-ART, a de novo assembly of the susceptible parental plant Adret2 was produced and compared to the recently established draft genome sequence of the resistant parental plant M2289 (Knorst et al. 2018). The sequencing of Adret2 resulted in 6.93E + 08 100 bp Illumina reads, which were filtered and assembled into 117,277 scaffolds with a minimum size of 2 kb. The total assembly size was 5.07E + 08 bp with an N50 value of 4760 bp (Table 1).

**Table 1:** Statistics of the sequencing and de novo assembly of the resistant (M2289) and susceptible (Adret2) Italian ryegrass parental plants of the Xtg-ART mapping population (Studer et al. 2006)

Parent	Number of bp in raw data	Coverage of raw reads^b^	Number of scaffolds > 2 kb	N50 scaffolds > 2 kb	Total assembly size (bp)
M2289^a^	6.89E + 10	27.56	129,579	4949	5.74E + 08
Adret2	6.93E + 10	27.72	117,277	4760	5.07E + 08

^a^Knorst et al. (2018)

^b^Assuming a genome size of 2.5E + 09 bp (Kopecký et al. 2010)

To assess the completeness of the gene space in the Adret2 de novo assembly, a BUSCO analysis using the Embryophyta lineage was performed (Simão et al. 2015). In total, 1440 BUSCO groups were searched and 75.5% were found to be complete in the Adret2 assembly, whereas 13.9% were recorded as missing (Table 2). Although the de novo assembly of M2289 had a slightly higher number of complete BUSCOs and a lower number of missing BUSCOs (80.5% and 11.0% compared to 75.5% and 13.9% in Adret2, respectively), both assemblies provide a solid basis for SNP discovery in the Xtg-ART mapping population.

**Table 2:** BUSCO (benchmarking universal single-copy orthologues) analysis of the de novo assemblies for the resistant (M2289) and susceptible (Adret2) parental Italian ryegrass plants of the Xtg-ART mapping population

Parent	Complete BUSCOs (%)	Complete and single-copy BUSCOs (%)	Complete and duplicated BUSCOs (%)	Fragmented BUSCOs (%)	Missing BUSCOs (%)	Total BUSCO groups searched
M2289^a^	80.5	59.7	20.8	8.5	11	1440
Adret2	75.5	64.8	10.7	10.6	13.9	1440

In total, 1440 BUSCO groups of the Embryophyta lineage were searched

^a^Knorst et al. (2018)

To identify SNPs segregating in the Xtg-ART mapping population (SegSNPs), the sequencing reads of M2289 and Adred2 were realigned to the more comprehensive assembly of M2289. In total, 18E + 06 SNPs heterozygous in at least one parent were extracted for further analysis.

Sequencing of the susceptible and resistant pools consisting of 44 *F*_1_ individuals each resulted in 1.55E + 08 and 1.53E + 08 100 bp reads, respectively. Reads from both pools were separately realigned to the M2289 de novo assembly and SNPs segregating in the population, as determined in the parental plants above, were extracted. A total of 12E + 06 of the previously identified SegSNPs (66%) had coverage with more than 20 reads per site and per pool. *F*_ST_ values of these SNPs were calculated and 271 SNPs on 140 different scaffolds exceeded an *F*_ST_ value of 0.9 (Supplementary Table 1), thereby being putatively associated with bacterial wilt resistance.

### Development and validation of molecular markers associated with bacterial wilt resistance

**Fig. 2:**
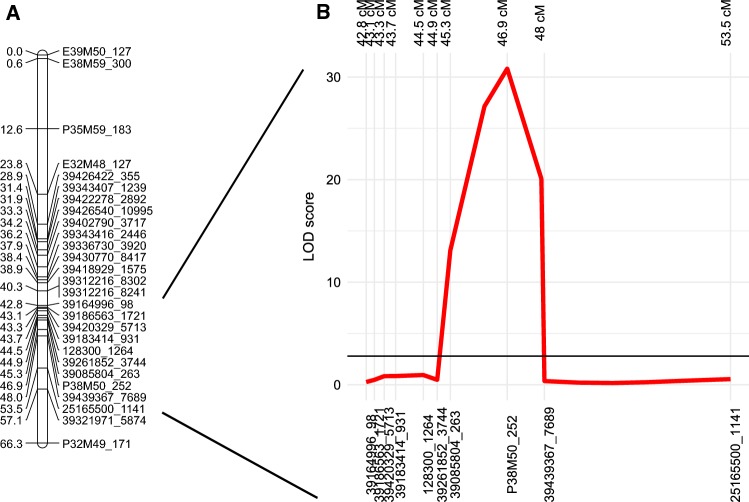
**a** Genetic linkage map of 20 scaffolds containing SNP markers identified by bulk segregant analysis (indicated by scaffold-number_SNP-position) and six previously reported AFLP markers surrounding the QTL region on linkage group 4 (E39M50_127, E38M59_300, P35M59_183, E32M48_127, P32M49_171) of the Italian ryegrass mapping population Xtg-ART as previously described by Studer et al. (2006). **b** Logarithm of the odds (LOD) score of the major QTL for bacterial wilt resistance on linkage group 4 of Italian ryegrass. The black line indicates the significance threshold of LOD 2.8

Of the 271 SNPs identified on 140 scaffolds, 30 SNPs on 29 scaffolds were selected for genotyping based on their putative assignment to LG 4 inferred by synteny with the draft genome assembly of perennial ryegrass (Byrne et al. 2015). These SNPs were genotyped in 221 *F*_1_ individuals of the Xtg-ART population using the KASP™ SNP genotyping technology. By single-marker linear regression analysis, 15 SNPs with *R*^2^ ≥ 0.2 for all the three disease score measurements reported in Studer et al. (2006) were identified, highlighting the association of these markers with bacterial wilt resistance (Table 3). SNP marker 39312216_8241 explained the largest proportion of variance for resistance in the glasshouse with *R*^2^ = 0.41, followed by SNP 39336730_3920 (*R*^2^ = 0.39). Together with eight additional markers, 13 out of these 15 SNPs mapped to a 28 cM interval on LG 4 of the Xtg-ART population, spanning the QTL region flanked by AFLP markers E32M48_127 and P32M49_171 (Studer et al. 2006; Fig. 2a). Association of these SNP markers with bacterial wilt resistance was further validated using QTL analysis and the disease scores from the previously reported glasshouse experiment (Studer et al. 2006). The major QTL for bacterial wilt resistance was located between 45 and 48 cM (Fig. 2b), with a maximum logarithm of odds (LOD) score of 30.8 at the location of the AFLP marker P38M50_252, while the SNP marker 39085804_263 was located 1.6 cM from the QTL peak. Another flanking marker, SNP 39439367_7689, failed to meet the LOD threshold but was located 1.1 cM from the QTL and had a highly significant association with resistance in the single-marker regression (*R*^2^ = 0.34; Table 3).

**Table 3:** Characteristics and results from single-marker regression of markers genotyped on 221 *F*_1_ progeny of the Xtg-ART mapping population using KASP™ assays

Marker characteristics					Single-marker regression					
Scaffold	SNP position	*F*_ST_ value	Allele	Annotation	Glasshouse		Field 2004		Field 2005	
					*R* ^*2*^	*P*	*R* ^*2*^	*P*	*R* ^*2*^	*P*
128300	1264	0.96	A/T	n.a.^a^	0.27	6.93E − 13	0.37	1.80E − 18	0.32	2.34E − 15
25165500	1141	1.00	C/G	AT4G38830; cystein-rich RLK 26	n.a.	n.a.	n.a.	n.a.	n.a.	n.a.
28703361	2476	0.98	A/T	n.a.	0.03	n.s.^b^	0.02	n.s.	0.02	n.s.
39085804	263	0.96	A/T	n.a.	0.35	3.43E − 22	0.29	5.59E − 18	0.34	8.61E − 22
39097100	1078	0.98	T/C	n.a.	0.02	n.s.	0.01	n.s.	0.02	n.s.
39107756	422	0.97	G/A	n.a.	0.19	5.93E − 08	0.18	5.26E − 08	0.33	4.19E − 14
39164996	98	1.00	C/T	n.a.	0.03	n.s.	0.03	n.s.	0.05	n.s.
39183414	931	0.96	G/A	n.a.	0.37	1.11E − 22	0.31	1.32E − 18	0.38	7.98E − 24
39186563	1721	0.96	T/C	n.a.	0.37	3.51E − 22	0.32	6.56E − 19	0.38	1.89E − 23
39261852	3744	0.96	G/A	n.a.	0.00	n.s.	0.01	n.s.	0.01	n.s.
39268474	886	0.97	C/A	n.a.	0.00	n.s.	0.01	n.s.	0.00	n.s.
39312216	8241	0.95	A/C	AT5G47850; CRINKLY4-related 4	0.41	4.42E − 26	0.30	1.38E − 18	0.40	2.50E − 25
39312216	8302	1.00	C/G	AT5G47850; CRINKLY4-related 4	0.33	4.80E − 20	0.26	2.64E − 15	0.34	2.26E − 20
39321971	5874	0.96	C/G	n.a.	0.18	1.43E − 10	0.15	5.44E − 09	0.19	1.94E − 11
39336730	3920	0.96	T/C	AT1G72030; Acyl-CoA n.a.T superfamily protein	0.39	5.85E − 25	0.29	1.37E − 17	0.39	4.27E − 25
39343407	1239	0.95	C/G	n.a.	0.14	1.12E − 06	0.13	2.03E − 06	0.18	1.46E − 08
39343416	2446	1.00	G/A	AT5G48380; BAK1 interacting RLK	n.a.	n.a.	n.a.	n.a.	n.a.	n.a.
39357736	2540	1.00	A/G	n.a.	0.00	6.00E − 01	0.01	n.s.	0.00	n.s.
39362400	3333	1.00	G/A	n.a.	0.34	8.15E − 20	0.20	7.69E − 12	0.29	3.52E − 17
39369100	2036	1.00	T/C	n.a.	0.00	9.46E − 01	0.00	n.s.	0.00	n.s.
39373787	2419	1.00	T/C	n.a.	0.27	4.78E − 12	0.26	3.34E − 12	0.27	1.38E − 12
39402790	3717	0.97	G/A	AT1G69550; TIR-NBS-LRR	n.a.	n.a.	n.a.	n.a.	n.a.	n.a.
39418929	1575	1.00	G/T	n.a.	0.37	1.42E − 22	0.30	1.03E − 17	0.40	1.00E − 24
39420329	5713	1.00	A/G	n.a.	0.36	1.08E − 22	0.29	6.83E − 18	0.37	3.05E − 23
39422278	2892	1.00	A/G	n.a.	0.33	1.09E − 19	0.20	6.11E − 12	0.28	9.93E − 17
39426422	355	0.97	G/A	AT3G07960; SWEET17	0.10	1.25E − 05	0.07	3.81E − 04	0.10	1.91E − 05
39426540	10,995	0.96	C/T	AT3G07960; Phosphatidylinositol-4-phosphate 5-kinase	0.24	5.92E − 15	0.22	9.90E − 14	0.28	2.06E − 17
39430715	4031	1.00	T/A	AT5G23530; carboxylesterase 18	0.20	5.66E − 10	0.13	1.33E − 06	0.20	3.19E − 10
39430770	8417	0.98	G/A	AT1G71140; MATE efflux family protein	0.23	1.31E − 13	0.20	3.77E − 12	0.24	2.64E − 14
39439367	7689	0.95	T/C	n.a.	0.34	1.13E − 20	0.24	3.14E − 14	0.32	1.44E − 19

*F*_ST_ was obtained by comparing allele frequencies in susceptible and resistant pools consisting of 44 progeny each. Scaffold numbers annotation refers to the reference genome of the *L. multiflorum* genotype M2289 (Knorst et al. 2018). Phenotypic data and the mapping population were previously reported by Studer et al. (2006)

^a^Not available

^b^Not significant (*P* > 0.01)

In order to simulate MAS markers with R^2^ > 0.2 in single-marker regression (Table 3) and mapping to LG 4 (Fig. 2) were selected. Positive single-marker MAS reduced the average disease score by up to 17% (marker 39439367_7689) when compared to the control without MAS (Table 4). Negative selection for the same marker increased the average disease score by 14% when compared to the control. Positive selection for combinations of two or three markers resulted in a decrease in the average disease score of up to 28% (markers 39430770_8417 & 39439367_7689) or 36% (markers 128300_1264 39430770_8417 & 39439367_7689), respectively, when compared to the no MAS control (Table 4).

**Table 4:** Simulation of marker-assisted selection (MAS) using selected KASP™ markers associated with bacterial wilt resistance and disease scores obtained in the greenhouse on 221 *F*_1_ progeny of the Xtg-ART mapping population (Studer et al. 2006)

Marker(s)	Positive selection			Negative selection		
	Mean	SD	N	Mean	SD	N
No MAS (control)	6.61^a^	1.57^a^	221^a^	n.a.^b^	n.a.^b^	n.a.^b^
*Single-marker MAS*						
39439367_7689	5.49	1.12	53	7.56	1.35	114
128300_1264	5.50	1.01	61	7.45	1.46	45
39312216_8302	5.66	1.16	94	7.48	1.38	118
39183414_931	5.67	1.17	116	7.97	0.87	4
39312216_8241	5.67	1.17	105	7.70	1.19	100
39186563_1721	5.71	1.24	116	7.65	1.23	89
39336730_3920	5.72	1.21	110	7.72	1.19	99
39418929_1575	5.74	1.23	114	7.70	1.22	94
39420329_5713	5.77	1.25	121	7.69	1.23	97
39085804_263	5.78	1.24	118	7.71	1.23	92
39422278_2892	5.83	1.27	116	7.68	1.26	91
39426540_10995	5.87	1.33	127	7.16	1.84	8
39430770_8417	6.01	1.37	51	7.63	1.16	91
*Two-marker MAS* ^*c*^						
39430770_8417 & 39439367_7689	4.75	0.71	2	7.69	1.17	83
128300_1264 & 39439367_7689	5.19	0.97	24	7.45	1.47	43
39312216_8302 & 39439367_7689	5.37	1.05	44	7.57	1.34	106
128300_1264 & 39312216_8302	5.38	0.91	49	7.43	1.48	41
39336730_3920 & 39439367_7689	5.38	1.01	50	7.79	1.14	89
*Three-marker MAS* ^*c*^						
128300_1264 & 39430770_8417 & 39439367_7689^d^	4.25	NA	1	7.55	1.29	34
39085804_263 & 39430770_8417 & 39439367_7689^d^	4.75	0.71	2	7.77	1.14	76
128300_1264 & 39312216_8302 & 39439367_7689	5.08	0.98	20	7.42	1.49	39
128300_1264 & 39186563_1721 & 39439367_7689	5.12	0.93	23	7.47	1.39	37
128300_1264 & 39336730_3920 & 39439367_7689	5.12	0.93	23	7.64	1.27	34

Three selection strategies were compared: no MAS, positive MAS (selection for homozygosity of the allele(s) associated with resistance) and negative MAS (selection for homozygosity of the alternative allele(s)). Mean denotes the mean disease score ranging from 1 (no symptoms) to 9 (plant dead), SD the standard deviation of the mean, and *N* the number of plants genotyped for the respective marker(s)

^a^No selection applied

^b^Not applicaple

^c^Only the first five marker combinations with the lowest mean value under positive selection are shown

^d^Only one of several combinations involving only one or two resistant individuals is shown

### Identification of candidate genes associated with bacterial wilt resistance

The 21 SNP markers mapping to LG 4 of Xtg-ART contained candidate genes for disease resistance. Of special interest were a gene for a CRINKLY4-related protein on scaffold 39312216, a gene coding for a nodulin MtN3 family protein (SWEET17) on scaffold 39426422, the BAK1-interacting receptor-like kinase 1 on scaffold 39343416, the cysteine-rich RLK (RECEPTOR-like protein kinase) 26 on scaffold 25165500 and the disease resistance protein (TIR-NBS-LRR class) on scaffold 39402790 (Table 3). Three of these four scaffolds contain more than one SNP with an *F*_ST_ value ≥ 0.9 in close proximity, stressing the potential importance of these scaffolds region (Supplementary Table 1). In addition, for two of the four scaffolds, SNP markers highly associated with bacterial wilt resistance were identified through single-marker regression (Table 3).

## Discussion

Here, we demonstrate the efficacy of NGS-based pooled DNA sequencing to enrich with sequence information a previously identified QTL region harboring an important source of bacterial wilt resistance in Italian ryegrass. The sequence information was utilized to develop and validate an inexpensive marker system that can now be used to fix resistance alleles in genetically heterogeneous breeding populations. Implementation of more efficient MAS-based breeding strategies to develop cultivars resistant to bacterial wilt is an important goal in one of the most important forage grass species worldwide. Furthermore, the candidate genes identified here provide interesting targets for further research, to increase the understanding of the complex interaction of Xtg and Italian ryegrass.

A major limitation in the development of MAS-based breeding strategies in forage grasses is the still rather limited availability of genomic sequence information and genome-wide marker resources. This is particularly true for allogamous and highly heterozygous forage grass species such as Italian ryegrass, which are characterized by large, highly complex and repetitive genomes (Byrne et al. 2015; Kopecky et al. 2006). While intensive sequencing efforts in perennial ryegrass (*L. perenne*) have led to the availability of a draft genome sequence (Byrne et al. 2015), such a resource has only recently become available for Italian ryegrass (Knorst et al. 2018). In the present study, this resource was complemented by a draft genome assembly of the susceptible parental plant (Adret2) of the Xtg-ART mapping population, where a large QTL accounting for up to 84% of the phenotypic variance for bacterial wilt resistance was previously identified (Studer et al. 2006). Although the two parental genomes of Xtg-ART are far from being complete, BUSCO values of more than 75% proved sufficient to give a first overview of the genome composition of the susceptible and resistant parent of this mapping family and, more importantly, to efficiently preselect a high number of SNPs segregating in the offspring. Based on the sequence data presented here, SNP calling produced about 1.8E + 07 SNPs being heterozygous in at least one parental plant. With an estimated assembly size of 5.74 × 10E + 08 bp, this corresponded to an average SNP density of 3.1 SNPs/100 bp of the assembly. This is in line with a previous study in perennial ryegrass, where 3.56 SNPs/100 bp in genic regions of perennial ryegrass were reported (Ruttink et al. 2015). In addition to the use in Xtg-ART, the sequence resources presented constitute an important source for genome-wide marker analysis in other Italian ryegrass genotypes and cultivars and will advance large-scale identification of species-specific SNPs in the *Festuca*–*Lolium* species complex (Birrer et al. 2014; Liu et al. 2018; Stočes et al. 2016).

BSA to link DNA markers to a trait of interest was first proposed in 1991 (Michelmore et al.) and has since been applied in forage grasses to determine linkage of the leafy head (*ldh 1*) mutation to AFLP markers in Italian ryegrass (Gao et al. 2002), to identify SSR or AFLP markers associated with resistance to crown rust (*Puccinia coronata* f. sp *lolii*) in perennial ryegrass (Dumsday et al. 2003; Fujimori et al. 2004; Muylle et al. 2005), to link EST-derived CAPS and AFLP markers to a gene conferring resistance to ryegrass blast caused by *Pyricularia* spp. (Miura et al. 2005) or to identify a novel major locus for gray leaf spot (*Magnaporthe oryzae*) resistance in Italian ryegrass (Takahashi et al. 2014). BSA has been shown to work best for traits controlled by a single locus or gene, causing a distinct phenotype that allows a correct pool assignment. An additional important success factor for BSA is a sufficiently high number of markers that are evenly distributed in the genome (Magwene et al. 2011). While in the above-mentioned studies a relatively low number of markers were used, more recently massive analysis of cDNA ends (MACE) allowed to identify a candidate gene for the *LpPg1* stem rust resistance in perennial ryegrass (Bojahr et al. 2016). A total of 145,707 SNPs identified between the pools of 20 resistant and 20 susceptible individuals provided a rich source of candidate SNPs co-segregating with stem rust resistance. Although the genetic resolution determined by recombination events around the causative gene in a total of 40 individuals was very limited, using a population of 276 individuals allowed mapping of 30 candidate transcripts to a segment of 50.8 cM, with the most closely linked markers surrounding the *LpPg1* locus at distances of 1.1 and 0.3 cM, respectively.

In the present study, we used whole genome sequencing of both pools to generate marker data at a very high density, even after filtering for SNPs that were heterozygous in at least one parental plant. Constructing the susceptible and the resistant pool with 44 individuals each enabled us to identify SNPs putatively associated with the major QTL for bacterial wilt resistance in Xtg-ART. We successfully converted 30 SNPs from 29 scaffolds to KASP™ markers and genetically mapped them using 221 Xtg-ART individuals. Strikingly, 21 of the selected SNP markers mapped within the interval spanned by the AFLP markers previously flanking the QTL region, clustering around the QTL peak at marker P38M50_252 (Studer et al. 2006). Furthermore, most of the 21 SNPs were significantly associated with bacterial wilt resistance, explaining up to 41% of the variance observed based on single-marker linear regression (Table 3). Thus, BSA was very efficient to identify trait-associated SNPs and enrich the target region with genomic sequence information. Given the relatively low number of individuals in both pools as well as the moderate number of individuals used for mapping and QTL analysis, we did not achieve a genetic resolution sufficiently high to pinpoint the causative gene conferring bacterial wilt resistance. However, we identified a set of biologically interesting candidate genes that can now be further analyzed in the genome of the susceptible and resistant parental plants of Xtg-ART. More importantly, the primary goal of this work was to develop molecular markers flanking the QTL for deployment in marker-assisted breeding strategies. Initial work to map the QTL in this population used AFLP markers, which are anonymous, dominant and not ideal for high-throughput analysis. Our approach enabled us to enrich the target QTL with sequence information, which could then be used to design KASP™ markers and select those flanking the QTL for breeding applications. KASP™ markers are ideal for breeding applications as they are inexpensive and amenable to high-throughput analysis (Semagn et al. 2014). KASP™-based assays have, for example, been used to develop breeder-friendly marker solutions for wheat leaf rust resistance (Neelam et al. 2013) or to accelerate public sector rice breeding (Steele et al. 2018). The KASP™ markers developed in this study have not only been shown to be significantly associated with bacterial wilt resistance (Table 3), and some of them also flank the QTL region previously identified (Fig. 2). Such markers may be particularly valuable for marker-assisted backcrossing (MAB; Frisch and Melchinger 2005) or marker-assisted recurrent selection (MARS; Bernardo and Charcosset 2006) to accelerate breeding for bacterial wilt resistance in ryegrasses. MAB has been shown to be particularly efficient in self-pollinating species such as rice where a large number of traits have been successfully introduced into elite germplasm using markers with varying levels of linkage to the target region (reviewed in Hasan et al. 2015). Ryegrasses are cross-pollinated species, usually bred as synthetic varieties based on multiparental crosses or improved as populations (Posselt 2010). In such species, MARS may be particularly valuable for population improvement using one or several cycles of a combination of marker-based and phenotypic selection (Bernardo and Charcosset 2006). In Italian ryegrass, a single SSR marker linked to crown rust resistance has been shown to considerably improve selection efficiency when compared to pure phenotypic selection (Kölliker et al. 2016).

In the present study, simulation of MAS within the same dataset, where the markers were identified in, showed the potential of increasing resistance in the population by selecting for resistance alleles using single markers or combinations of two or three markers (Table 4). Due to the limited population size, individuals homozygous for resistance alleles at three-marker loci were rather scarce. However, twenty individuals homozygous for resistance alleles were identified for the combination of markers 128300_1264, 39312216_8302, 39439367_7689 and average disease score among these individuals was with 5.08 considerably lower when compared to the mean of the entire population (6.61; Table 4). Thus, the markers identified have a high potential for application of MAS in resistance breeding programs, but they need to be validated in different genetic backgrounds.

Among the scaffolds mapping to the QTL region, a number of interesting candidate genes were found. On scaffold 39312216, for example, a gene for CRINKLY4 (serine/threonine protein kinase-like protein CCR4)-related protein was identified. A CRINKLY4-related kinase was found to be upregulated in *Brassica napus* after infection with *Sclerotinia sclerotiorum* and functioned as a pathogen recognition receptor (PRR) leading to pathogen-associated molecular pattern (PAMP) triggered immunity (PTI) after pathogen detection (Wu et al. 2016). Thus, in the Xtg-Italian ryegrass interaction, CRINKLY4 might be involved in the detection of Xtg and the initiation of PTI.

A gene coding for a nodulin MtN3 family protein, also known as SWEET17 (Chardon et al. 2013), was annotated for scaffold 3942642. In rice, SWEET13 encodes for *xa25* and confers resistance against *X. oryzae* pv. *oryzae* (Xoo; Cheng et al. 2017). SWEET transporters seem to be a target of vascular pathogens to allocate sugars to promote their growth (Chen 2014). As Xtg is a vascular pathogen primarily invading the xylem of the host plant (Masuch et al. 1989), sugars might be transported into the apoplast as an energy source for Xtg and a mutation in the sugar transporter may lead to plant resistance based on the limited availability of energy. However, in rice, Xoo activates SWEET via TALEs (transcription activator-like effectors) which are missing in Xtg (Wichmann et al. 2013).

Scaffold 39343416 encoded for the protein BAK1 (BRI1 [Brassinosteroid Insensitive 1]-associated kinase-interacting receptor-like kinase 1). BAK1 is involved in PTI and forms a heterodimer with FLS2 (flagellin sensing 2) after recognition of the bacterial derived PAMP flg22 (Chinchilla et al. 2007; Schulze et al. 2010). Another scaffold, 39420287, carrying two SNPs with *F*_ST_ values of 0.96 and 0.97, encodes for a transthyretin-like protein which is a potential substrate for BRI1 (Nam and Li 2004). This suggests that there is a similar recognition mode of Xtg by Italian ryegrass as it is known for *A. thaliana* and the detection of a conserved region of the bacterial flagellum, flg22 (Chinchilla et al. 2006). However, the Xtg strains sequenced so far all lack the flagellar gene cluster (Hersemann et al. 2017). Thus, these observed candidate genes may present remainders from the arms race between host and pathogen, and the genes for recognition might still be present in Italian ryegrass as part of a cluster of resistance genes (Sekhwal et al. 2015).

Other candidate genes identified included a cysteine-rich RLK (RECEPTOR-like protein kinase) 26 known to be induced after pathogen attack (scaffold 25165500; Chen et al. 2004) or a disease resistance protein (TIR-NBS-LRR class) with no reported specific function (scaffold 39402790; Meyers et al. 2003). In summary, the candidate genes identified through BSA and QTL mapping include PRRs involved in PTI as well as classical R genes involved in effector-triggered immunity ETI.

In conclusion, we successfully used a combination of genome sequencing and BSA to identify SNP markers associated with Xtg resistance in Italian ryegrass and enrich a major QTL with DNA sequence information. This enabled us to design and validate an inexpensive molecular marker system for use in marker-assisted breeding applications aimed at increasing the frequency of the resistance QTL in breeding populations. We envisage being able to use these markers through marker-assisted recurrent selection and to substantially increase selection efficiency for bacterial wilt resistance in Italian ryegrass.

### Author contribution statement

VK obtained and analyzed the data and drafted the manuscript. SB and SY performed genome assembly and annotation and helped drafting the manuscript. TA and BS helped with data analysis and interpretation and improving the manuscript. RK conceived the study, assisted data analysis and interpretation of the results and drafted the manuscript. All authors read and approved the final version of the manuscript.

## Electronic supplementary material

Below is the link to the electronic supplementary material.

Supplementary material 1 (XLSX 32 kb)
